# Lima LV, Pavinati G, Moroskoski M, Gil NLM, Takenami IO, Magnabosco GT. Inequalities in the provision of rapid HIV testing in primary health care: ecological study, Paraná, 2013-2023. Epidemiol Serv Saude. 2026:35;e20250981

**DOI:** 10.1590/S2237-96222026v35e20250981.a

**Published:** 2026-10-12

**Authors:** José Nildo de Barros Silva Júnior

**Affiliations:** 1 Fundação Oswaldo Cruz, Rio de Janeiro, RJ, Brazil Fundação Oswaldo Cruz Rio de Janeiro RJ Brazil

## Peer review A

## Questionnaire

Does the manuscript contain new and significant information to justify publication? Yes

Does the Abstract (Summary) clearly and accurately describe the content of the article? Yes

Is the problem significant and concisely stated? Yes

Are the methods described comprehensively? Yes

Are the interpretations and conclusions justified by the results? Yes

Is adequate reference made to other work in the field? Yes

## Manuscript Structure

Length of article is: Adequate

Number of tables is: Adequate

Number of figures is: Adequate

Please state any conflict(s) of interest that you have in relation to the review of this paper. None

Interest: Excellent

Quality: Good

Originality: Good

Overall: Good

Recommendation: Minor Revision

## Comments

Prezados autores,

O estudo enviado apresenta informações relevantes para a saúde pública e para a ciência, com boa escrita e método bem justificado. Na avaliação, buscou-se identificar os pontos que merecem maior atenção, a fim de qualificar ainda mais o manuscrito.

TÍTULO:

Adequado.

RESUMO:

- No método, foi utilizado o termo “indicados”, que provavelmente deveria ser “indicadores”.

- Considerando que o objetivo apresenta três componentes (temporal, espacial e fatores associados), espera-se que os resultados façam menção clara a esses três aspectos. No entanto, o componente espacial não é abordado de forma explícita.

- Da mesma forma, a conclusão deve responder aos três componentes do objetivo, mas atualmente enfatiza majoritariamente os fatores associados.

INTRODUÇÃO:

- O termo Atenção Primária à Saúde deve ser padronizado com iniciais maiúsculas, por se tratar de um conceito formal definido pela Organização Mundial da Saúde e pelo Ministério da Saúde do Brasil.

- O último parágrafo da página 2 necessita de uma citação.

- O estado da arte requer aprimoramento. Existem outros estudos que avaliaram a descentralização do teste rápido para HIV na APS no Brasil? Este é o primeiro realizado com dados do Paraná? Qual lacuna do conhecimento o estudo busca preencher em relação à literatura já publicada sobre a temática? É importante deixar clara, na problematização, a necessidade de conduzir um estudo com os três componentes propostos (temporal, espacial e fatores associados) para uma compreensão mais ampla do fenômeno.

- Na afirmação “Seus resultados podem subsidiar estratégias que ampliem o acesso ao diagnóstico precoce e fortalecer o papel da APS no enfrentamento ao HIV no Brasil”, recomenda-se esclarecer por que os autores extrapolam as conclusões para o âmbito nacional, considerando que o estudo tem foco estadual. Seria interessante contextualizar o papel da organização e da tomada de decisão no próprio estado.

- A sigla aids deve ser escrita por extenso na primeira vez em que aparecer no texto.

MÉTODO:

- Na descrição das variáveis, recomenda-se deixar claro de qual sistema cada uma foi extraída. Inicialmente, os autores o fazem ao mencionar as variáveis provenientes do SAI, mas posteriormente isso não é mantido. Sugere-se uma nova leitura dessa seção, garantindo que todas as fontes estejam devidamente descritas, seja no texto ou em uma tabela com colunas indicando as variáveis e o sistema de origem.

- Incluir informações sobre os aspectos éticos, esclarecendo a não necessidade de submissão ao CEP.

RESULTADOS:

- Recomenda-se que os autores avaliem a questão da variação percentual anual (VPA). Nenhum dos locais apresentou quebra na série histórica? Caso contrário, a avaliação de toda a série sem observar as quebras corresponderia à Variação Percentual Anual Média (VPAM). Sugere-se revisar este ponto. Além disso, recomenda-se sinalizar com um asterisco (*) os resultados estatisticamente significativos, ou apresentá-los em negrito, para facilitar a leitura da Tabela 1.

DISCUSSÃO:

- Na página 7, linhas 39 a 42. Seria interessante aprofundar a discussão sobre “levando a uma priorização da oferta em serviços especializados”, considerando que a recomendação prioritária é a realização na APS. E o estudo também não avaliou o contraponto com o serviço especializado. Sugere-se trazer evidências de outros estudos, realizados em diferentes contextos nacionais, e evitar encerramentos genéricos, como a frase “[...] para mitigar essas desigualdades.” Recomenda-se uma nova leitura para tornar os conceitos e argumentos mais claros e específicos.

- O parágrafo da página 7, linhas 45 a 52, carece de referências. Além disso, a explicação apresentada, de que o problema se deve apenas a lacunas na formação e capacitação, parece simplificada. Sugere-se aprofundar a análise e inserir referências adequadas.

- Senti falta de uma seção ou parágrafo final com perspectivas futuras para novos estudos, o que poderia enriquecer a discussão.

